# Coping strategies for managing diabetes distress in adults with type 1 and type 2 diabetes: a cross-sectional study on use and perceived usefulness

**DOI:** 10.3389/fcdhc.2024.1462196

**Published:** 2024-11-08

**Authors:** Jiska Embaye, Frank Jan Snoek, Maartje de Wit

**Affiliations:** Department of Medical Psychology, Amsterdam UMC location Vrije Universiteit Amsterdam, Amsterdam Public Health, Amsterdam, Netherlands

**Keywords:** type 1 diabetes, type 2 diabetes, chronic disease, distress, coping, self-management

## Abstract

**Purpose:**

The purpose of this study is to identify the use of coping strategies employed by adults with type 1 and type 2 diabetes to manage diabetes distress, using data provided by persons with lived experiences.

**Methods:**

Adults with diabetes completed an anonymous online survey on Diabetes.co.uk, describing their coping strategies. A follow-up survey assessed the frequency of use and perceived usefulness of these strategies. Statistical analyses, including Mann-Whitney U tests, compared strategy use and usefulness between participants with low vs. high diabetes distress.

**Results:**

625 adults with T1D or T2D completed the survey (mean age 56.3 years; 58.9% were female). Problem-focused strategies, “Taking care of my diabetes” and “Eating healthy,” were most frequently used and perceived as useful. Emotion-focused strategies such as “Expressing my emotions (crying or being angry)” were less used and perceived less useful. Participants with low vs. diabetes distress showed differences in strategy use.

**Conclusions:**

Adults with T1D and T2D use various coping strategies for diabetes distress, with problem-focused coping being more common and found useful than emotion-focused coping. Providing individuals with a list of effective coping strategies can enhance their awareness and adoption of new strategies. Integrating personalized coping strategies into interventions can better support diabetes management.

## Introduction

Upon being diagnosed with a chronic condition such as diabetes, individuals are confronted with a new life that requires adjustments. Adjustment, as described by De Ridder and colleagues (1), refers to healthy rebalancing by people to their new circumstances, which involves the physical, psychological and social domains (2). Examples of adjustments in diabetes are managing daily routines such as monitoring blood glucose levels throughout the day, dietary regulation and insulin administration, as well as navigating the emotional challenges associated with diabetes, managing interpersonal relationships and seeking support. The demands of living with diabetes can impose a significant burden and translate into emotional distress (3). Emotional distress in people with diabetes, also known as diabetes distress, is reported by approximately 30% of adults with diabetes (4–6). Elevated diabetes distress has been associated with suboptimal self-management, in terms of diet, exercise and medication adherence, negatively impacting glycaemic outcomes (7–10). Whereas lower levels of diabetes distress have been linked to better diabetes self-management and glycaemic outcomes (11), where cause and effect are difficult to discern.

Healthy coping plays an important role in adjusting to the demands of living with a chronic condition like diabetes. Over the years, a vast body of research has examined coping strategies in chronic disease (1, 12, 13) including diabetes. In diabetes particular, studies have shown that healthy coping is associated with lower levels of distress (14), enhanced diabetes self-care (15), and improved glycaemic outcomes (16). The Association of Diabetes Care and Education Specialists (ADCES) defines healthy coping as “a positive attitude towards diabetes and self-management, positive relationships with others, and quality of life” (11). They have proposed seven key self-care behaviours that are necessary for effective self-management. Healthy coping is emphasized by the ADCES as the cornerstone for mastering the other six behaviours (healthy eating, being active, taking medication, monitoring, reducing risk and problems solving).

Lazarus and Folkman’s stress-coping theory provides a framework to understand differences in adjustment outcomes in chronic disease (17). Lazarus and Folkman argued that the experience of stress differs significantly between individuals depending on how they interpret an event. According to this framework stress arises when a certain event is perceived as threatening or harmful and coping refers to how individuals manage threats posed by a stressor. Coping has been hypothesized to be an intervening variable between perceptions of stressors (appraisal) and adjustment (18). Lazarus and Folkman distinguished two primary dimensions of coping. Problem-focused coping involves actively addressing the stressor directly, such as in the case of diabetes by focusing on managing the blood glucose regulation (monitoring blood glucose levels, administering insulin), or seeking information on diabetes, and obtaining support from healthcare professionals. Emotion-focused coping aims to address the emotional distress that results from the stressful event. This approach entails a wide range of strategies, including suppressive strategies such as suppression of emotions or acceptance, and expressive strategies as venting of emotions (17). Problem-focused coping has more often been linked to positive health outcomes, such as improved glycaemic outcomes and dietary behaviour (19) and better adjustment overall in people with diabetes (18). However, in situations where stressors become overwhelming and difficult to control, as sometimes observed in challenges related to managing diabetes, emotion-focused coping may become more suitable. For instance, when self-managing diabetes becomes distressing, using effective strategies to cope with these negative emotions becomes essential. Therefore, coping flexibility is also of importance, which is defined as ‘one’s ability to modify one’s coping strategies adaptively to meet the demands of different stressful situations’ (20), and this is associated with more adaptive outcomes.

In addition to the traditional categories of problem-focused and emotion-focused coping, other strategies have also been suggested. One strategy is proactive coping which is closely related to problem-focused coping. It is described as “the efforts people undertake in advance of a potentially stressful event to prevent or modify its form before it occurs (21). This future-oriented approach is crucial for individuals with diabetes because effective self-management can prevent potential dysregulation, physical symptoms, and long-term complications (7–10). And effective self-management can help reduce emotional distress (11). Additional categories include avoidance coping, which involves strategies aimed at distancing from the stressor (e.g. suppression of activities, denial, disengagement, self-distraction), often associated with greater diabetes distress (22). Cognitive-focused coping strategies (e.g. cognitive reappraisal) are also recognized. Furthermore, other types of coping strategies can be identified such as spirituality, religion or seeking for support (23–25). Both social support and hope have been found to be important for coping with diabetes and its emotional challenges (26).

Given the critical role of healthy coping in managing diabetes, this study aimed to explore the coping strategies used by adults with type 1 and type 2 diabetes in addressing diabetes distress. Specifically, we aimed to (1) identify and rank the frequency and perceived usefulness of these coping strategies, (2) investigate differences in the rankings between individuals with low versus high diabetes distress, and (3) examine the variation in the ‘active’ use of coping strategies between these two groups. Whereas previous studies on coping with diabetes typically relied on pre-existing coping scales, this study used data from adults with lived experiences to asses coping (15, 23, 27, 28).

## Methods

### Participants and procedure

Before the present study, an exploratory investigation was conducted, in which no previous data analysis had been performed on this dataset, nor had any prior publications been produced. In this investigation, adults living with diabetes were invited to complete an anonymous online survey hosted on Diabetes.co.uk, a community platform where individuals with diabetes connect to share support, insights, and experiences. Participants were requested to describe strategies they use to cope with diabetes distress in response to an open-ended question. During this stage, a total of 653 participants, (387 individuals with type 2 diabetes, 255 with type 1 diabetes, 8 with LADA, 2 with MODY and 1 with gestational diabetes; majority between 45-64 years old) provided responses to the open-ended question. Examples of responses to the open-ended question include “A day with my four year olds”, “Getting out as much as possible”, “Going to gym when I have the energy”, “Block thoughts out” and “Being able to discuss how and what you feel”. Two researchers independently categorized and labelled these strategies based on overarching themes eliminating strategies that can be perceived as harmful (such as overeating and substance abuse), which resulted in a final set of 46 strategies. Examples of the refined strategies from the final list include: “Using humour”, “Practising a hobby” and ´ Avoiding stressful stimuli”. **Supplementary Tables 1** and **2** provide a full overview of all 46 coping strategies ranked from most to least used and most to least perceived as useful strategies). With a follow-up anonymous survey on Diabetes.co.uk, the frequency and usefulness of these 46 strategies to manage diabetes distress was assessed, as well as clinical and demographic variables and level of diabetes distress. All individuals living with any type of diabetes were included in the study, but only responses of adults with T1D or T2D were used for the present study. Fluency in English was necessary to participate due to the survey’s language, and access to a mobile device or computer was required for online participation. No additional exclusion criteria were applied. All participants consented to take part in the study.

### Measures

The following demographic and clinical data were measured through self-report: age, sex, ethnicity, level of education, marital status, living status, type of diabetes, the year of diabetes diagnosis, most recent A1C, and the occurrence of recent severe hypoglycaemic episodes. Additionally, participants were asked if they had ever received professional psychological help, and whether they were taking medication for stress, depression or anxiety.

Participants were invited to evaluate the frequency with which they employ each of the 46 strategies, using a five-point Likert scale from 0 (never) to 4 (always). Subsequently, participants rated the perceived usefulness of each strategy on a Likert scale from 0 (not useful at all) to 4 (extremely useful). If a participant indicated never to use a specific strategy, the usefulness was not assessed.

The 5-item Problem Areas in Diabetes Scale-Short Form-5 (PAID-5) was used to measure diabetes-related distress. Participants rank their problems on a 5-point Likert scale (ranging from 0 for “not a problem” to 4 for “serious problem”), with higher scores indicating greater diabetes distress. A score of 8 is used as a cut-off for elevated diabetes distress (29).

### Statistical analyses

Firstly, descriptive statistics were used to describe the populations demographic characteristics and diabetes-related characteristics, using mean and standard deviation, or frequencies and percentages in the case of categorical data. Then, descriptive statistics were used to assess the frequency and perceived usefulness of the 46 coping strategies. For each strategy, a total mean score was calculated, and the strategies were ranked accordingly. This ranking was further analysed in relation to PAID-5 scores, comparing groups with PAID-5 scores <8 versus those with scores ≥8. Additionally, the distribution of responses for each coping strategy was examined by determining the percentage of participants scoring at the lowest (0) or highest (4) possible levels on the Likert scale.

To assess the differences in use and usefulness of coping strategies between PAID-5 < 8 and PAID-5 ≥ 8, two Mann-Whitney U tests were conducted. This test was chosen due to the non-normal distribution of the data, as assessed by the Shapiro-Wilk test (p < 0.05). A Mann-Whitney U test does not allow for controlling of covariates. Prior to the Mann-Whitney U test, descriptive statistics were calculated for both groups and mean scores and standard deviations were reported. A Bonferroni correction was applied to account for multiple comparisons across 46 variables, resulting in a new significance level of α = 0.05/46 = α = 0.001. Effect sizes were calculated from the Z-scores of the U-tests, with the following formula: r = z/√N. Cohens’ d effect size interpretation for standardized scores was used in which < 0.2 = small, 0.2 - 0.5 = medium, and 0.8 > is a large effect size (30).

Lastly, to examine the differences in ‘active’ use of a strategy between PAID-5 < 8 and PAID-5 ≥ 8, which relates to coping flexibility, a Mann-Whitney U test was performed. For each participant, the total number of actively used strategies (defined as a score of 3 or higher on a scale of 0-4) was calculated. Adults scoring PAID-5 < 8 were expected to show higher mean scores on active strategy use.

The analyses were conducted with SPSS statistics version 28.

## Results

### Descriptive statistics

Out of 1258 people who joined the online survey, 625 adults (50%) with either T1D or T2D completed the whole survey. **Table 1** outlines the demographic and clinical characteristics. The mean age of the participants was 56.3 years (SD=14.5), the majority were female (58.9%), white (93.1%), and higher educated (69.5%). Approximately 43.5% reported elevated diabetes distress (PAID-5 ≥8), while 4.2% were currently receiving professional psychological support, 20.6% had a history of professional psychological support and 75.2% had never received any type of professional psychological support. Additionally, 19.8% of participants was currently taking medication for stress, depression or anxiety.

**Table 1:** Baseline characteristics of study population.

Characteristics mean (SD) or %	
Total, N	625
Age in years, range, mean (SD)	56.3 (14.5)
Female, n (%)	367 (58.9)
Transgender/ non-binary	1 (0.16)
Ethnicity: White, n (%)	582 (93.1)
Married/partner, n (%)	441 (70.6)
Employed, n (%)	309 (46.2)
Education: bachelor’s degree or higher	244 (69.5)
Diabetes duration in years, mean (SD) (n=563)	15 (15.7)
Diabetes type, n (%)	
Type 1	256 (40.9)
Type 2	369 (59)
Type 2 with insulin therapy	130 (35.2)
Type 2 without insulin therapy	239 (64.8)
A1C, mmol/mol mean (SD) (n=588)	53.2 (16.2)
PAID-5 total, mean (SD)	7.4 (5.6)
PAID-5 ≥8, n (%)	272 (43.5)

The group who exhibited elevated diabetes distress (PAID-5 ≥8), more often consisted of people with T1D (55.5% vs. 29.7%), (χ²(1, N = 625) = 42.187, p <.001). This group more often comprised females (63.6% vs. 55.1%) (χ²(1, N = 625) = 4.565, p = 0.033). On average this group was younger (M=50.1 vs. M=61.1 years, (U = 20181, p <.001, r = 0.49)). Additionally they reported higher A1C levels (57.8 mmol/mol vs. 49.8 mmol/mol, U = 280004, p <.001, r = 0.28)).

### Ranking of frequency of use and usefulness of coping strategies based on means

Overall, there appears to be consensus regarding the ranking of the frequency and usefulness of coping strategies, as displayed in **Tables 2**, **3**, showing the five most and least used strategies. **Supplementary Tables 1** and **2** provide a comprehensive list of all 46 coping strategies ranked from most to least used and most to least perceived as useful strategies, including the percentage distribution of individuals who rated each strategy.

**Table 2:** Top 5 most used and useful coping strategies, for the total sample.

Ranking	Frequency of use, scale 0-4				Usefulness, scale 0-4			
		n	Mean	SD		n	Mean	SD
1	Taking care of my diabetes (checking blood glucose level, taking medication)	625	3.21	1.1	Taking care of my diabetes (checking blood glucose level, taking medication)	597	3.2	1.04
2	Eating healthy/ responsibly or dieting	625	2.9	1	Eating healthy/ responsibly or dieting	608	2.9	1.1
3	Spending time with family and friends	625	2.6	1.1	Having good health care providers	538	2.9	1.2
4	Standing up for myself	625	2.6	1.2	Taking some time for myself	578	2.7	1.1
5	Having a routine	625	2.5	1.2	Going outside/ getting fresh air	575	2.7	1.2

**Table 3:** Top 5 least used and useful coping strategies, for the total sample.

Ranking	Frequency of use, scale 0-4				Usefulness, scale 0-4			
		n	Mean	SD		n	Mean	SD
1	Doing structured attention exercises (yoga, meditation, mindfulness, breathing exercises)	625	0.90	1.15	Tracking my mood	382	1.74	1.18
2	Using an antidepressant	625	0.73	1.40	Explaining to others	510	1.62	1.12
3	Practicing religious activities	625	0.70	1.28	Going into therapy (e.g. cognitive behaviour therapy, coaching)	108	1.51	1.21
4	Writing (in diary of blog)	625	0.62	1.12	Asking support from my surroundings	370	1.44	1.12
5	Going into therapy (e.g. cognitive behaviour therapy. coaching)	625	0.27	0.68	Expressing my emotions (crying or being angry)	454	1.34	1.16

The rankings of use and perceived usefulness of coping strategies, highlights two problem-focused strategies that are frequently used and also considered most useful: “Taking care of my diabetes” emerges as the most frequently employed strategy, with only 4.5% reporting never using it, and it’s also perceived as the most useful, with 55.7% rating it as extremely useful. “Eating healthy/responsibly or dieting” follows as the second most frequently used strategy, with only 2.7% reporting never using it, and it’s the second most perceived as useful, with 39.7% considering it extremely useful.

Interestingly, “Asking support from my surroundings” is seldom used, ranking 40th in frequency, with 40.8% of individuals never using this strategy. Among those who do use it, it ranks as the second least useful, with 20.5% rating it as not useful at all. Similarly, “Expressing my emotions (crying or being angry),” an emotion-focused strategy, ranks relatively low in frequency of use (38th), with 27.4% never employing this strategy. Among those who do, it is rated as the least useful, with 29.3% finding it not useful at all. Lastly, “Tracking my mood” is ranked 39^th^ in frequency of use, with 38.9% never using this strategy. It also ranks low in perceived usefulness, at 43^rd^ place, with 14.7% indicating that they did not find it useful at all.

Further, there are strategies that see little frequency of use but are considered relatively useful by those who employ them: “Practising religious activities (for example praying, going to church)” ranks 44^th^ in frequency of use, with 71.2% of participants never using this strategy. Despite its infrequent use, it ranks 24^th^ in perceived usefulness, with 26.7% rating it as extremely useful. Likewise, “Having contact with others who go through the same experience” ranks 41st in frequency of use, with 44% never employing this strategy. However, it ranks relatively higher in perceived usefulness, placing 33rd, with 12.9% rating it as extremely useful.

There were no differences observed in ranking of the strategies between individuals with T1D and individuals with T2D.

### Coping strategies: low vs. high diabetes distress

**Supplementary Tables 3** and **4** present the rankings for frequency of use and perceived usefulness of all strategies among individuals with low versus high diabetes distress. No large differences were found between the groups in terms of ranking: in both cases, problem-focused strategies ranked highest, while emotion-focused strategies were at the bottom.

However, there were differences between frequency of use and perceived usefulness. **Tables 4**, **5** demonstrate the results of the strategies that showed significant differences between those with elevated vs. low diabetes distress (PAID-5 ≥ 8 vs. PAID-5 < 8), demonstrating small to medium effect sizes.

**Table 4:** Low vs. high diabetes distress: significant differences in frequency of use of coping strategies.

Coping strategy	PAID-5 < 8 (n=353)		PAID-5 ≥ 8 (n=272)		U	p	r
	Mean	SD	Mean	SD			
Asking support from my surroundings	0.88	0.98	1.28	1.14	38427	p<.001	0.18
Being busy with my job or work related activities	1.78	1.37	2.17	1.33	40445	p= .001	0.14
Caring for someone or something else	1.64	1.39	2.12	1.35	38674	p<.001	0.17
Choosing what I tell to whom	2.17	1.33	2.83	1.08	34521	p<.001	0.25
Comparing my situation with others who are worse off than me	1.72	1.35	2.07	1.30	41027	p= .001	0.13
Doing something that distracts me from my thoughts	1.82	1.20	2.26	1.01	37804	p<.001	0.19
Eating healthy/ responsibly or dieting	3.10	0.97	2.57	1.03	33545	p<.001	0.27
Expressing my emotions (crying or being angry)	1.15	1.10	1.79	1.16	33358	p<.001	0.27
Going into therapy (e.g. cognitive behavioural therapy, coaching)	0.14	0.46	0.43	0.85	40813	p<.001	0.20
Positive thinking/ optimism	2.54	1.25	1.97	1.15	34699	p<.001	0.24
Practicing a hobby	1.88	1.35	2.00	1.18	40947	p= .001	0.13
Seeing the positive side of diabetes	2.46	1.12	1.17	1.20	33631	p<.001	0.26
Taking some time for myself	0.45	1.10	2.16	1.11	40510	p= .001	0.14
Using an antidepressant	0.88	0.98	1.10	1.64	38579	p<.001	0.22

**Table 5:** Low vs. high diabetes distress: significant differences in perceived usefulness of coping strategies.

Coping strategy	PAID-5 < 8			PAID-5 ≥ 8			U	p	r
	n	Mean	SD	n	Mean	SD			
Doing something that distracts me from my thoughts	287	2.15	1.14	253	2.47	1.12	30305	p= .001	0.14
Eating healthy/ responsibly or dieting	345	3.13	1.03	263	2.66	1.21	35473	p<.001	0.20
Having contact with others who go through the same experience	213	1.92	1.02	137	2.40	1.13	11026	p<.001	0.21
Positive thinking/ optimism	319	2.72	1.16	239	2.38	1.15	31755	p<.001	0.15
Using an antidepressant	60	1.93	1.42	253	2.47	1.12	1941	p<.001	0.28

Participants, on average, actively employed 16.5 coping strategies (SD = 9, range 0 – 43). No significant differences were observed between respondents reporting low vs. high diabetes distress (M = 15.94, SD = 8.61) and (M = 16.93, SD = 9.29) in terms of active use (U = 44586, p = 0.126, r = 0.06).

Examples of strategies in which differences were observed are “Doing something that distracts me from my thoughts”, which was more often used by participants with elevated diabetes distress (U = 37804, p <.001, r = 0.19) and more often found useful (U = 33545, p <.001, r = 0.27). Also “Expressing my emotions (crying or being angry)” was more often used by participants with elevated diabetes distress (U = 33358, p <.001, r = 0.27) and more often found useful (U = 30305, p = .001, r = 0.14). “Eating healthy/responsibly or dieting” was more often used by participants with low diabetes distress (U = 33545, p <.001, r = 0.27) and more often found useful (U = 33545, p <.001, r = 0.27). Similarly, “Positive thinking/optimism”, was more often used by those with low diabetes distress (U = 34699, p <.001, r = 0.24) and more often found useful (U = 31755, p <.001, r = 0.15).

## Discussion

This study examined coping strategies employed by adults with T1D and T2D to manage diabetes-related distress. The reported use of coping strategies revealed that problem-focused strategies, directly addressing the management of diabetes and the maintenance of a healthy lifestyle (also perceived as proactive coping), were both the most frequently used and perceived as the most useful. This underscores the ongoing challenge faced by individuals with diabetes: the need to achieve a balance between managing blood glucose levels to prevent both immediate and long-term complications, while also leading a fulfilling life (31). Conversely, strategies associated with seeking therapy and emotion-focused strategies were least used and perceived as least useful.

Overall, there was a consensus between the ranking of the use and usefulness of coping strategies, with few exceptions: “Practising religious activities” and “Having contact with others who go through the same experience” seemed to be very personal strategies that were favoured in use by only a select group of adults, but nevertheless perceived as relatively useful by those who employed them.

For most strategies, there were no differences in use, perceived usefulness, or ranking of coping strategies between individuals with low and high diabetes distress. However, “Positive thinking/optimism” was less often used by those with elevated diabetes distress than by those with low diabetes distress. This aligns with earlier findings that optimistic beliefs are associated with lower levels of distress, anxiety and depression, and improved physical functioning in chronic disease (32). Additionally, the strategy “Eating healthy/responsibly or dieting” was less frequently used and considered less useful by those with elevated diabetes distress. This seems in line with previous findings that elevated diabetes distress is associated with suboptimal self-management (7–10). Moreover, the strategies “Doing something that distracts me from my thoughts” and “Expressing my emotions (crying or being angry),” also known as venting (referring to the expression of unpleasant or negative feelings), can be viewed as less adaptive coping strategies (33). These were more frequently used by participants with elevated diabetes distress, in line with existing literature that finds self-distraction and venting to be associated with increased diabetes distress and depression (23).

It is important to note that the group who exhibited elevated diabetes distress more often consisted of females, had a younger average age, more often had T1D, and had higher A1C levels. These in particular demographic factors could also explain differences that were found in use of coping strategies. For example, our results showed that “Expressing my emotions (crying or being angry)” was more often used by those with elevated diabetes distress, however sex could have also played a role in this finding as women more often tend to use emotion-focused coping and men more often problem-focused coping (34). Although there are inconsistencies in findings, it has been suggested that women with T2D more often use resignation, protest and isolation (less adaptive coping strategies) (26). Other studies find that men with diabetes more often use active coping, less avoidant coping, and less support-seeking than women with diabetes (12). Furthermore, research on aging suggests that older adults cope better with stress because their past experiences in coping with stress have led to better emotion regulation strategies (35, 36), but literature in the context of diabetes is lacking. Religious and spiritual coping are more common in cultures where this plays a larger role (24, 25).

Contrary to our expectations, we did not find higher scores for active strategy use among those without elevated diabetes distress. Instead, both groups, those with and without elevated diabetes distress, actively employed numerous coping strategies on average. This may be due to the fact that the strategies were highly varied, making it difficult to draw conclusions on coping flexibility based solely on the number of strategies actively used.

A strength of this study is its large and balanced sample size, with 41.1% of participants being male. Another strength lies in the coping list that was used: unlike most studies that rely on pre-existing coping lists (27), we developed a comprehensive list of 46 coping strategies based on input from individuals with lived experiences. This approach ensured that the coping strategies examined in our study were relevant and comprehensive, reflecting the diverse range of strategies individuals use in real-life situations.

Limitations include the retrospective cross-sectional design of this study, which limits the ability to measure the context in which specific coping strategies are used and perceived as useful and makes it difficult to draw conclusions. Future studies could include additional assessment measures, such as *ad hoc* semi-structured interviews, to qualitatively explore the coping strategies individuals use in various situations and how useful they perceive them to be, particularly in the context of diabetes-related distress. Adopting a qualitative research perspective would also enable researchers to focus on individual experiences.

Additionally, the cross-sectional design of the study makes it challenging to determine the relationship between coping strategies and diabetes distress. Higher distress scores might lead individuals to adopt certain coping strategies, but it is also possible that using certain coping strategies over an extended period could lead to higher distress scores. Based on the results of this study it is not possible to say that the use of certain strategies directly translates in lower levels of distress. Longitudinal studies would provide a better understanding of the direction of this association. Furthermore, while the PAID-5 questionnaire measures diabetes distress at the present moment, the coping questionnaire asked participants about the strategies they have used or found useful without specifying the time frame over which these strategies were employed.

Furthermore, this study included only ‘adaptive’ or ‘healthy’ coping strategies in the generation of the list of 46 coping strategies, as we aimed to use the results for interventions. However, this decision means that an opportunity was missed to compare the use of these strategies with potentially less adaptive ones.

Lastly, the sampling method may have caused bias, as only members of the Diabetes.co.uk community were eligible to participate, who are likely more actively engaged in their diabetes than the broader diabetes population Furthermore, the sample consisted predominantly of individuals of white ethnicity and with higher education levels, limiting the generalizability.

## Conclusion

Adults with T1D and T2D use a wide variety of coping strategies for dealing with diabetes distress. Problem-focused coping strategies, specifically those aimed at addressing diabetes-related challenges, are more frequently used and found more useful than emotion-focused strategies.

Diabetes distress is prevalent and providing individuals with diabetes a comprehensive list of potentially effective coping strategies could help increase their awareness of their own use of coping strategies to deal with distress and inspire them to explore and adopt new strategies. Furthermore, it is important for healthcare providers to consider the individual’s perspective and the context in which they use specific strategies, rather than categorizing strategies as inherently “good” or “bad”. Coping is a dynamic process that can vary over time, across different contexts, and among individuals of different sexes, ages, and cultures. Therefore, it is important to consider the context and intersections of these aspects when studying coping. By integrating these individualized coping strategies into interventions, healthcare providers can better support individuals with diabetes in effectively managing diabetes-related distress (37). Moreover, these recommended strategies can be included in self-help interventions for adults with diabetes, aimed at improving healthy coping (38).

## Data Availability

The raw data supporting the conclusions of this article can be made available by the authors upon request.
