# Supplementary material for: Coping strategies for managing diabetes distress in adults with type 1 and type 2 diabetes: a cross-sectional study on use and perceived usefulness
Source: Front Clin Diabetes Healthc. 2024 Nov 8;5:1462196. doi: 10.3389/fcdhc.2024.1462196 (PMC11582030; doi:10.3389/fcdhc.2024.1462196)
Supplement: Supplementary file 1 [file Table1.docx]

**Supplement 1a. Coping strategies ranked by mean frequency of use scores (n=625, Likert Scale 0-4)**

| **Ranking** | **Coping strategy** | **Mean** | **SD** | **0 = never, n (%)** | **4 = always, n (%)** |
| --- | --- | --- | --- | --- | --- |
|  | Taking care of my diabetes (checking blood glucose level. taking medication) | 3.21 | 1.11 | 28 (4.5%) | 348 (55.7%) |
|  | Eating healthy/ responsibly or dieting | 2.87 | 1.03 | 17 (2.7%) | 203 (32.5%) |
|  | Spending time with family and friends | 2.62 | 1.15 | 42 (6.7%) | 154 (24.6%) |
|  | Standing up for myself | 2.56 | 1.22 | 49 (7.8%) | 167 (26.7%) |
|  | Having a routine | 2.54 | 1.19 | 49 (7.8%) | 149 (23.8%) |
|  | Going outside/ getting fresh air | 2.46 | 1.16 | 50 (8%) | 119 (19%) |
|  | Choosing what I tell to whom | 2.46 | 1.27 | 73 (11.7%) | 153 (24.5%) |
|  | Doing low intensity exercise (walking. cycling) | 2.39 | 1.26 | 65 (10.4%) | 137 (21.9%) |
|  | Searching for information about diabetes and/ or stress | 2.38 | 1.20 | 61 (9.8%) | 111 (17.8%) |
|  | Using humour | 2.34 | 1.17 | 55 (8.8%) | 116 18.6%) |
|  | Taking some time for myself | 2.33 | 1.12 | 47 (7.5%) | 97 (15.5%) |
|  | Positive thinking/ optimism | 2.29 | 1.24 | 67 (10.7%) | 124 (19.8%) |
|  | Showing love and gratitude | 2.29 | 1.18 | 64 (10.2%) | 104 (16.6%) |
|  | Going to sleep or rest | 2.28 | 1.09 | 48 (7.7%) | 79 (12.6%) |
|  | Having good health care providers | 2.25 | 1.30 | 87 (13.9%) | 127 (20.3%) |
|  | Thinking I’m not the only one | 2.25 | 1.28 | 92 (14.7%) | 111 (17.8%) |
|  | Practising a hobby | 2.17 | 1.27 | 87 (13.9%) | 111 (17.8%) |
|  | Making plans for the future | 2.14 | 1.25 | 88 (14.1%) | 97 (15.%) |
|  | Thinking about something nice/ pleasant | 2.13 | 1.12 | 68 (10.9%) | 63 (10.1%) |
|  | Distraction through enjoyable activities | 2.11 | 1.17 | 79 (12.6%) | 65 (10.4%) |
|  | Change thoughts into something positive | 2.09 | 1.21 | 89 (14.2%) | 78 (12.5%) |
|  | Doing something that distracts me from my thoughts | 2.01 | 1.14 | 85 (13.6%) | 50 (8%) |
|  | Being busy with my job or work related activities | 1.95 | 1.37 | 147 (23.5%) | 81 (13%) |
|  | Breaking my diabetes management down into manageable chunks (prioritizing. planning) | 1.91 | 1.35 | 133 (21.3%) | 89 (14.2%) |
|  | Talking with my health care provider(s) | 1.88 | 1.18 | 93 (14.9%) | 66 (10.6%) |
|  | Comparing my situation with others who are worse off than me | 1.87 | 1.34 | 140 (22.4%) | 80 (12.8%) |
|  | Sharing and getting information via forum(s) or social media | 1.86 | 1.25 | 121 (19.4) | 63 (10.1%) |
|  | Sort out what’s causing the stress and what influence it has on my body and mind. | 1.86 | 1.17 | 106 (17%) | 42 (6.7%) |
|  | Talking about diabetes and related issues or feelings with my significant others. | 1.85 | 1.27 | 120 (19.2%) | 68 (10.9%) |
|  | Caring for someone or something else | 1.85 | 1.39 | 163 (26.1%) | 83 (13.3%) |
|  | Exercising/ doing sport activities | 1.84 | 1.31 | 123 (19.7%) | 86 (13.8%) |
|  | Explaining to others | 1.74 | 1.14 | 115 (18.4%) | 38 (6.1%) |
|  | Organizing activities | 1.72 | 1.22 | 133 (21.3%) | 50 (8%) |
|  | Avoiding stressful stimuli | 1.64 | 1.13 | 132 (21.1%) | 27 (4.3%) |
|  | Seeing the positive side of diabetes | 1.57 | 1.33 | 187 (29.9%) | 67 (10.7%) |
|  | Reading about positive experiences | 1.54 | 1.16 | 154 (24.6%) | 33 (5.3%) |
|  | Having a mantra/ positive self-encouragement | 1.51 | 1.43 | 232 (37.1%) | 72 (11.5%) |
|  | Expressing my emotions (crying or being angry) | 1.43 | 1.17 | 171 (27.4%) | 29 (4.6%) |
|  | Tracking my mood | 1.15 | 1.15 | 243 (38.9%) | 24 (3.8%) |
|  | Asking support from my surroundings | 1.05 | 1.07 | 255 (40.8%) | 14 (2.2%) |
|  | Having contact with others who go through the same experience | 0.99 | 1.12 | 275 (44%) | 25 (4%) |
|  | Doing structured attention exercises (yoga, meditation, mindfulness, breathing exercises) | 0.90 | 1.15 | 329 (52.6%) | 20 (3.2%) |
|  | Using an antidepressant | 0.73 | 1.40 | 469 (70.6%) | 30 (4.8%) |
|  | Practising religious activities | 0.70 | 1.28 | 445 (71.2%) | 55 (8.8%) |
|  | Writing (in diary of blog) | 0.62 | 1.12 | 441 (70.6%) | 30 (4.8%) |
|  | Going into therapy (e.g. cognitive behaviour therapy. coaching) | 0.27 | 0.68 | 517 (82.7%) | 3 (0.5%) |
