# Supplementary material for: Coping strategies for managing diabetes distress in adults with type 1 and type 2 diabetes: a cross-sectional study on use and perceived usefulness
Source: Front Clin Diabetes Healthc. 2024 Nov 8;5:1462196. doi: 10.3389/fcdhc.2024.1462196 (PMC11582030; doi:10.3389/fcdhc.2024.1462196)
Supplement: Supplementary file 2 [file Table2.docx]

**Supplement 1b. Coping strategies ranked by mean usefulness scores (Likert Scale 0-4)**

| **Ranking** | **Coping strategy** | **N** | **Mean** | **SD** | **0 = not useful at all, n (%)** | **4 = extremely useful, n (%)** |
| --- | --- | --- | --- | --- | --- | --- |
|  | Taking care of my diabetes (checking blood glucose level, taking medication) | 597 | 3,22 | 1.04 | 14 (2.3%) | 319 (53.4%) |
|  | Eating healthy/ responsibly or dieting | 608 | 2.93 | 1.13 | 20 (3.2%) | 248 (39.7%) |
|  | Having good health care providers | 538 | 2.87 | 1.17 | 21 (3.9%) | 211 (33.8%) |
|  | Taking some time for myself | 578 | 2.74 | 1.10 | 19 (3%) | 169 (27%) |
|  | Going outside/ getting fresh air | 575 | 2.70 | 1.16 | 21 (3.7%) | 172 (27.5%) |
|  | Spending time with family and friends | 583 | 2.67 | 1.18 | 27 (4.6%) | 177 (28.3%) |
|  | Having a routine | 576 | 2.67 | 1.16 | 25 (4.3%) | 169 (27%) |
|  | Going to sleep or rest | 577 | 2.58 | 1.16 | 26 (4.5%) | 145 (23.2%) |
|  | Positive thinking/ optimism | 558 | 2.58 | 1.16 | 21 (3.8%) | 148 (23.7%) |
|  | Doing low intensity exercise (walking, cycling) | 560 | 2.54 | 1.26 | 38 (6.8%) | 159 (28.4%) |
|  | Using humour | 570 | 2.50 | 1.15 | 25 (4.4%) | 134 (23.5%) |
|  | Standing up for myself | 576 | 2.49 | 1.22 | 36 (6.3%) | 147 (25.5%) |
|  | Distraction through enjoyable activities | 546 | 2.47 | 1.14 | 26 (4.8%) | 116 (21.2%) |
|  | Searching for information about diabetes and/ or stress (via internet, course, or health care providers) | 564 | 2.45 | 1.17 | 25 (4.4%) | 130 (23%) |
|  | Using an antidepressant | 156 | 2.44 | 1.38 | 15 (9.6%) | 51 (32.7%) |
|  | Practising a hobby | 538 | 2.44 | 1.20 | 36 (6.7%) | 122 (22.7%) |
|  | Exercising/ doing sport activities | 502 | 2.43 | 1.30 | 45 (9%) | 138 (27.5%) |
|  | Talking with my health care provider(s) | 532 | 2.40 | 1.20 | 32 (6%) | 120 (22.6%) |
|  | Making plans for the future | 537 | 2.32 | 1.15 | 30 (5.6%) | 95 (17.7%) |
|  | Showing love and gratitude | 561 | 2.30 | 1.19 | 39 (7%) | 107 (19.1%) |
|  | Doing something that distracts me from my thoughts | 540 | 2.30 | 1.14 | 34 (6.3%) | 81 (15%) |
|  | Thinking about something nice/ pleasant | 557 | 2.29 | 1.18 | 38 (6.8%) | 99 (17.8%) |
|  | Sort out what’s causing the stress and what influence it has on my body and mind. | 519 | 2.25 | 1.14 | 32 (6.2%) | 78 (15%) |
|  | Practising religious activities (for example praying, going to church) | 180 | 2.21 | 1.40 | 21 (11.7%) | 48 (26.7%) |
|  | Talking about diabetes and related issues or feelings with my significant others. | 505 | 2.18 | 1.19 | 41 (8.1%) | 82 (16.2%) |
|  | Sharing and getting information via forum(s) or social media | 504 | 2.18 | 1.18 | 37 (7.3%) | 80 (15.9%) |
|  | Reading about positive experiences | 471 | 2.17 | 1.10 | 29 (6.2%) | 60 (12.7%) |
|  | Change thoughts into something positive | 536 | 2.17 | 1.18 | 42 (7.8%) | 80 (14.9%) |
|  | Breaking my diabetes management down into manageable chunks (prioritizing, planning) | 492 | 2.14 | 1.17 | 39 (7.9%) | 63 (12.8) |
|  | Thinking I’m not the only one | 533 | 2.13 | 1.28 | 65 (12.2%) | 92 (17.3%) |
|  | Having a mantra/ positive self-encouragement (e.g. “Master diabetes or it will master me” or “I’m doing the best that I can”) | 393 | 2.11 | 1.32 | 46 (11.7%) | 79 (201.%) |
|  | Having contact with others who go through the same experience | 350 | 2.11 | 1.09 | 15 (4.3%) | 45 12.9%) |
|  | Seeing the positive side of diabetes | 438 | 2.08 | 1.20 | 41 (9.4%) | 59 13.5%) |
|  | Organizing activities | 492 | 2.08 | 1.16 | 44 (8.9%) | 66 (13.4%) |
|  | Writing (in diary of blog) | 184 | 2.07 | 1.26 | 16 (8.7%) | 34 (18.5%) |
|  | Being busy with my job or work related activities | 478 | 2.01 | 1.21 | 61 (12.8%) | 46 (11.7%) |
|  | Choosing what I tell to whom | 552 | 1.99 | 1.23 | 65 (11.8%) | 74 (13.4%) |
|  | Avoiding stressful stimuli | 493 | 1.97 | 1.16 | 48 (9.7%) | 52 (10.5%) |
|  | Doing structured attention exercises (yoga. Meditation, mindfulness. breathing exercises) | 296 | 1.94 | 1.27 | 44 (14.9%) | 42 (14.2%) |
|  | Caring for someone or something else | 462 | 1.94 | 1.14 | 51 (11%) | 37 (8%) |
|  | Comparing my situation with others who are worse off than me | 485 | 1.88 | 1.31 | 86 (17.7%) | 68 (17.7%) |
|  | Tracking my mood | 382 | 1.74 | 1.18 | 56 (14.7%) | 36 (9.4%) |
|  | Explaining to others | 510 | 1.62 | 1.12 | 83 (16.3%) | 36 (7.1%) |
|  | Going into therapy (e.g. cognitive behaviour therapy, coaching) | 108 | 1.51 | 1.21 | 25 (23.1%) | 7 (6.5%) |
|  | Asking support from my surroundings | 370 | 1.44 | 1.12 | 76 (20.5%) | 17 (4.6%) |
|  | Expressing my emotions (crying or being angry) | 454 | 1.34 | 1.16 | 133 (29.3%) | 19 (4.2%) |
