# Supplementary material for: Coping strategies for managing diabetes distress in adults with type 1 and type 2 diabetes: a cross-sectional study on use and perceived usefulness
Source: Front Clin Diabetes Healthc. 2024 Nov 8;5:1462196. doi: 10.3389/fcdhc.2024.1462196 (PMC11582030; doi:10.3389/fcdhc.2024.1462196)
Supplement: Supplementary file 3 [file Table3.docx]

**Supplement 2a. Coping strategies ranked by mean frequency of use scores, by PAID scores (n=625, Likert Scale 0-4).**

|  | **PAID-5<8 (n=353)** | | | **PAID-5≥8 (n=272)** | | |
| --- | --- | --- | --- | --- | --- | --- |
| **Ranking** | **Coping strategy** | **Mean** | **SD** | **Coping strategy** | **Mean** | **SD** |
| 1. | Taking care of my diabetes (checking blood glucose level. taking medication) | 3.30 | 1.20 | Taking care of my diabetes (checking blood glucose level. taking medication) | 3.1 | 1.00 |
| 2. | Eating healthy/ responsibly or dieting | 3.1 | 1.00 | Choosing what I tell to whom | 2.8 | 1.10 |
| 3. | Spending time with family and friends | 2.7 | 1.20 | Eating healthy/ responsibly or dieting | 2.6 | 1.00 |
| 4. | Standing up for myself | 2.7 | 1.20 | Spending time with family and friends | 2.6 | 1.10 |
| 5. | Having a routine | 2.5 | 1.30 | Having a routine | 2.5 | 1.10 |
| 6. | Positive thinking/ optimism | 2.54 | 1.25 | Going outside/ getting fresh air | 2.43 | 1.07 |
| 7. | Going outside/ getting fresh air | 2.48 | 1.22 | Standing up for myself | 2.42 | 1.22 |
| 8. | Taking some time for myself | 2.46 | 1.12 | Going to sleep or rest | 2.40 | 1.02 |
| 9. | Doing low intensity exercise (walking. cycling) | 2.43 | 1.29 | Doing low intensity exercise (walking. cycling) | 2.34 | 1.21 |
| 10. | Searching for information about diabetes and/ or stress | 2.41 | 1.19 | Searching for information about diabetes and/ or stress | 2.33 | 1.21 |
| 11. | Using humour | 2.37 | 1.22 | Using humour | 2.31 | 1.09 |
| 12. | Showing love and gratitude | 2.32 | 1.21 | Having good health care providers | 2.27 | 1.24 |
| 13. | Practising a hobby | 2.30 | 1.32 | Doing something that distracts me from my thoughts | 2.26 | 1.01 |
| 14. | Thinking I’m not the only one | 2.25 | 1.33 | Thinking I’m not the only one | 2.26 | 1.22 |
| 15. | Having good health care providers | 2.24 | 1.35 | Showing love and gratitude | 2.24 | 1.14 |
| 16. | Making plans for the future | 2.22 | 1.26 | Distraction through enjoyable activities | 2.21 | 1.05 |
| 17. | Thinking about something nice/ pleasant | 2.20 | 1.16 | Being busy with my job or work related activities | 2.17 | 1.33 |
| 18. | Change thoughts into something positive | 2.19 | 1.27 | Taking some time for myself | 2.16 | 1.11 |
| 19. | Going to sleep or rest | 2.18 | 1.14 | Caring for someone or something else | 2.12 | 1.35 |
| 20. | Choosing what I tell to whom | 2.17 | 1.33 | Comparing my situation with others who are worse off than me | 2.07 | 1.30 |
| 21. | Distraction through enjoyable activities | 2.04 | 1.24 | Thinking about something nice/ pleasant | 2.04 | 1.05 |
| 22. | Breaking my diabetes management down into manageable chunks (prioritizing. planning) | 1.99 | 1.40 | Making plans for the future | 2.04 | 1.23 |
| 23. | Sort out what’s causing the stress and what influence it has on my body and mind. | 1.89 | 1.24 | Practising a hobby | 2.00 | 1.18 |
| 24. | Talking with my health care provider(s) | 1.89 | 1.21 | Positive thinking/ optimism | 1.97 | 1.15 |
| 25. | Sharing and getting information via forum(s) or social media | 1.89 | 1.27 | Change thoughts into something positive | 1.97 | 1.11 |
| 26. | Seeing the positive side of diabetes | 1.88 | 1.35 | Talking about diabetes and related issues or feelings with my significant others. | 1.86 | 1.22 |
| 27. | Exercising/ doing sport activities | 1.88 | 1.36 | Talking with my health care provider(s) | 1.86 | 1.15 |
| 28. | Talking about diabetes and related issues or feelings with my significant others. | 1.85 | 1.30 | Sharing and getting information via forum(s) or social media | 1.83 | 1.22 |
| 29. | Doing something that distracts me from my thoughts | 1.82 | 1.20 | Explaining to others | 1.82 | 1.14 |
| 30. | Being busy with my job or work related activities | 1.78 | 1.37 | Sort out what’s causing the stress and what influence it has on my body and mind. | 1.81 | 1.07 |
| 31. | Organizing activities | 1.75 | 1.26 | Breaking my diabetes management down into manageable chunks (prioritizing. planning) | 1.81 | 1.27 |
| 32. | Comparing my situation with others who are worse off than me | 1.72 | 1.35 | Exercising/ doing sport activities | 1.79 | 1.25 |
| 33. | Explaining to others | 1.68 | 1.14 | Expressing my emotions (crying or being angry) | 1.79 | 1.16 |
| 34. | Caring for someone or something else | 1.64 | 1.39 | Avoiding stressful stimuli | 1.78 | 1.08 |
| 35. | Reading about positive experiences | 1.59 | 1.16 | Organizing activities | 1.69 | 1.17 |
| 36. | Having a mantra/ positive self-encouragement | 1.56 | 1.52 | Reading about positive experiences | 1.46 | 1.16 |
| 37. | Avoiding stressful stimuli | 1.53 | 1.15 | Having a mantra/ positive self-encouragement | 1.45 | 1.31 |
| 38. | Tracking my mood | 1.16 | 1.21 | Asking support from my surroundings | 1.28 | 1.14 |
| 39. | Expressing my emotions (crying or being angry) | 1.15 | 1.10 | Seeing the positive side of diabetes | 1.17 | 1.20 |
| 40. | Having contact with others who go through the same experience | 1.06 | 1.09 | Tracking my mood | 1.14 | 1.08 |
| 41. | Asking support from my surroundings | 0.88 | 0.98 | Using an antidepressant | 1.10 | 1.64 |
| 42. | Doing structured attention exercises (yoga. meditation. mindfulness. breathing exercises) | 0.87 | 1.15 | Doing structured attention exercises (yoga. meditation. mindfulness. breathing exercises) | 0.93 | 1.15 |
| 43. | Practising religious activities | 0.82 | 1.38 | Having contact with others who go through the same experience | 0.90 | 1.15 |
| 44. | Writing (in diary of blog) | 0.64 | 1.16 | Writing (in diary of blog) | 0.58 | 1.05 |
| 45. | Using an antidepressant | 0.45 | 1.10 | Practising religious activities | 0.54 | 1.13 |
| 46. | Going into therapy (e.g. cognitive behaviour therapy. coaching) | 0.14 | 0.46 | Going into therapy (e.g. cognitive behaviour therapy. coaching) | 0.43 | 0.85 |
