# Supplementary material for: Coping strategies for managing diabetes distress in adults with type 1 and type 2 diabetes: a cross-sectional study on use and perceived usefulness
Source: Front Clin Diabetes Healthc. 2024 Nov 8;5:1462196. doi: 10.3389/fcdhc.2024.1462196 (PMC11582030; doi:10.3389/fcdhc.2024.1462196)
Supplement: Supplementary file 4 [file Table4.docx]

**Supplement 2b. Coping strategies ranked by mean usefulness scores (Likert Scale 0-4). categorized by PAID scores**

| **Ranking** | **PAID<8** | | | | **PAID-5≥8** | | | |
| --- | --- | --- | --- | --- | --- | --- | --- | --- |
|  |  | n | Mean | SD |  | n | Mean | SD |
|  | Taking care of my diabetes (checking blood glucose level. taking medication) | 331 | 3.3 | 1.00 | Taking care of my diabetes (checking blood glucose level. taking medication) | 266 | 3.1 | 1.10 |
|  | Eating healthy/ responsibly or dieting | 345 | 3.1 | 1.00 | Having good health care providers | 240 | 3.00 | 1.10 |
|  | Having good health care providers | 298 | 2.8 | 1.20 | Using an antidepressant | 96 | 2.8 | 1.30 |
|  | Going outside/ getting fresh air | 319 | 2.8 | 1.10 | Taking some time for myself | 251 | 2.7 | 1.10 |
|  | Taking some time for myself | 327 | 2.8 | 1.10 | Having a routine | 257 | 2.7 | 1.10 |
|  | Positive thinking/ optimism | 319 | 2.72 | 1.16 | Spending time with family and friends | 254 | 2.67 | 1.16 |
|  | Doing low intensity exercise (walking. cycling) | 309 | 2.67 | 1.23 | Eating healthy/ responsibly or dieting | 263 | 2.66 | 1.21 |
|  | Spending time with family and friends | 329 | 2.67 | 1.19 | Going outside/ getting fresh air | 256 | 2.59 | 1.16 |
|  | Having a routine | 319 | 2.66 | 1.19 | Going to sleep or rest | 260 | 2.59 | 1.16 |
|  | Using humour | 317 | 2.58 | 1.18 | Doing something that distracts me from my thoughts | 253 | 2.47 | 1.12 |
|  | Standing up for myself | 325 | 2.58 | 1.23 | Distraction through enjoyable activities | 250 | 2.45 | 1.12 |
|  | Going to sleep or rest | 317 | 2.56 | 1.17 | Searching for information about diabetes and/ or stress (via internet. course. or health care providers) | 242 | 2.41 | 1.16 |
|  | Exercising/ doing sport activities | 277 | 2.55 | 1.25 | Practising a hobby | 235 | 2.41 | 1.19 |
|  | Distraction through enjoyable activities | 296 | 2.49 | 1.15 | Having contact with others who go through the same experience | 137 | 2.40 | 1.13 |
|  | Searching for information about diabetes and/ or stress (via internet. course. or health care providers) | 322 | 2.48 | 1.18 | Using humour | 253 | 2.40 | 1.11 |
|  | Practising a hobby | 303 | 2.46 | 1.21 | Standing up for myself | 251 | 2.39 | 1.20 |
|  | Talking with my health care provider(s) | 298 | 2.42 | 1.21 | Doing low intensity exercise (walking. cycling) | 251 | 2.39 | 1.27 |
|  | Making plans for the future | 305 | 2.37 | 1.15 | Positive thinking/ optimism | 239 | 2.38 | 1.15 |
|  | Showing love and gratitude | 315 | 2.36 | 1.20 | Talking with my health care provider(s) | 234 | 2.38 | 1.19 |
|  | Thinking about something nice/ pleasant | 311 | 2.29 | 1.20 | Thinking about something nice/ pleasant | 246 | 2.30 | 1.16 |
|  | Change thoughts into something positive | 301 | 2.25 | 1.22 | Exercising/ doing sport activities | 225 | 2.28 | 1.35 |
|  | Practising religious activities (for example praying. going to church) | 113 | 2.25 | 1.44 | Sort out what’s causing the stress and what influence it has on my body and mind. | 234 | 2.27 | 1.13 |
|  | Sort out what’s causing the stress and what influence it has on my body and mind. | 285 | 2.24 | 1.14 | Making plans for the future | 232 | 2.26 | 1.16 |
|  | Breaking my diabetes management down into manageable chunks (prioritizing. planning) | 275 | 2.20 | 1.18 | Showing love and gratitude | 246 | 2.22 | 1.19 |
|  | Talking about diabetes and related issues or feelings with my significant others. | 280 | 2.19 | 1.13 | Sharing and getting information via forum(s) or social media | 220 | 2.20 | 1.16 |
|  | Having a mantra/ positive self-encouragement (e.g. “Master diabetes or it will master me” or “I’m doing the best that I can”) | 213 | 2.19 | 1.35 | Reading about positive experiences | 197 | 2.18 | 1.10 |
|  | Thinking I’m not the only one | 293 | 2.18 | 1.24 | Talking about diabetes and related issues or feelings with my significant others. | 225 | 2.18 | 1.26 |
|  | Reading about positive experiences | 274 | 2.17 | 1.10 | Choosing what I tell to whom | 259 | 2.14 | 1.22 |
|  | Sharing and getting information via forum(s) or social media | 284 | 2.16 | 1.19 | Practising religious activities (for example praying. going to church) | 67 | 2.13 | 1.34 |
|  | Doing something that distracts me from my thoughts | 287 | 2.15 | 1.14 | Organizing activities | 220 | 2.13 | 1.14 |
|  | Seeing the positive side of diabetes | 276 | 2.13 | 1.22 | Writing (in diary of blog) | 81 | 2.11 | 1.26 |
|  | Being busy with my job or work related activities | 256 | 2.05 | 1.23 | Breaking my diabetes management down into manageable chunks (prioritizing. planning) | 217 | 2.07 | 1.16 |
|  | Organizing activities | 272 | 2.04 | 1.17 | Change thoughts into something positive | 235 | 2.07 | 1.12 |
|  | Writing (in diary of blog) | 103 | 2.03 | 1.26 | Thinking I’m not the only one | 240 | 2.06 | 1.32 |
|  | Caring for someone or something else | 241 | 1.97 | 1.17 | Having a mantra/ positive self-encouragement (e.g. “Master diabetes or it will master me” or “I’m doing the best that I can”) | 180 | 2.02 | 1.29 |
|  | Avoiding stressful stimuli | 262 | 1.97 | 1.17 | Doing structured attention exercises (yoga. meditation. mindfulness. breathing exercises) | 136 | 2.00 | 1.26 |
|  | Using an antidepressant | 60 | 1.93 | 1.42 | Seeing the positive side of diabetes | 162 | 1.99 | 1.15 |
|  | Having contact with others who go through the same experience | 213 | 1.92 | 1.02 | Avoiding stressful stimuli | 231 | 1.97 | 1.16 |
|  | Comparing my situation with others who are worse off than me | 259 | 1.92 | 1.32 | Being busy with my job or work related activities | 222 | 1.96 | 1.20 |
|  | Doing structured attention exercises (yoga. meditation. mindfulness. breathing exercises) | 160 | 1.89 | 1.28 | Caring for someone or something else | 221 | 1.91 | 1.10 |
|  | Choosing what I tell to whom | 293 | 1.86 | 1.22 | Comparing my situation with others who are worse off than me | 226 | 1.85 | 1.31 |
|  | Tracking my mood | 210 | 1.71 | 1.17 | Tracking my mood | 172 | 1.78 | 1.20 |
|  | Explaining to others | 284 | 1.64 | 1.16 | Going into therapy (e.g. cognitive behaviour therapy. coaching) | 69 | 1.72 | 1.17 |
|  | Asking support from my surroundings | 186 | 1.36 | 1.07 | Explaining to others | 226 | 1.58 | 1.08 |
|  | Expressing my emotions (crying or being angry) | 224 | 1.24 | 1.15 | Asking support from my surroundings | 184 | 1.51 | 1.16 |
|  | Going into therapy (e.g. cognitive behaviour therapy. coaching) | 39 | 1.13 | 1.20 | Expressing my emotions (crying or being angry) | 230 | 1.44 | 1.17 |
